# Optical Biosensors Based on Semiconductor Nanostructures

**DOI:** 10.3390/s90705149

**Published:** 2009-06-29

**Authors:** Raúl J. Martín-Palma, Miguel Manso, Vicente Torres-Costa

**Affiliations:** Departamento de Física Aplicada and CIBER bbn, Universidad Autónoma de Madrid, 28049 Cantoblanco, Madrid, Spain

**Keywords:** nanostructure, quantum dot, biosensor

## Abstract

The increasing availability of semiconductor-based nanostructures with novel and unique properties has sparked widespread interest in their use in the field of biosensing. The precise control over the size, shape and composition of these nanostructures leads to the accurate control of their physico-chemical properties and overall behavior. Furthermore, modifications can be made to the nanostructures to better suit their integration with biological systems, leading to such interesting properties as enhanced aqueous solubility, biocompatibility or bio-recognition. In the present work, the most significant applications of semiconductor nanostructures in the field of optical biosensing will be reviewed. In particular, the use of quantum dots as fluorescent bioprobes, which is the most widely used application, will be discussed. In addition, the use of some other nanometric structures in the field of biosensing, including porous semiconductors and photonic crystals, will be presented.

## 1. Introduction

The increasing availability of techniques for the fabrication and characterization of semiconductor-based nanometric structures with controlled composition and dimensions has sparked widespread interest aiming at their use in different biotechnological systems [1,2], including biosensors [3,4]. Moreover, the precise control over the size, shape and composition of semiconductor nanostructures leads to the accurate control of their physico-chemical properties, thus allowing tailoring their response. Additionally, modifications can be made to the nanostructures to better suit their integration with biological systems, leading to such interesting properties as enhanced aqueous solubility, biocompatibility or bio-recognition.

Furthermore, the typical size of semiconductor nanostructures, comparable to that of many common biomolecules, makes them to be appropriate for the development of hybrid systems [5]. With selected biomolecules bound to nanostructure surfaces, new hybrid nanostructures can be obtained for optical biosensing and imaging. However, the idea of merging biological and non-biological systems at the nanoscale is not a new one. The broad field of bioconjugate chemistry is based on combining the functionalities of biomolecules and non-biological molecular species for specialized use in various different applications. Many current applications of nanostructures in biotechnology are a natural evolution of this approach. In fact, several of the most recently demonstrated applications using nanostructure–biomolecular hybrids are in fact traditional ones originally addressed by standard molecular bioconjugate techniques that have been revisited with newly designed nanostructure hybrids.

The interest in the replacement of conventional molecular tags, such as fluorescent chromophores, with nanostructures resides in the superior physico-chemical properties of nanostructures compared to the molecular species they replace [6,7]. These include issues such as higher quantum efficiencies, greater scattering or absorbance cross sections, optical activity over more biocompatible wavelengths and significantly increased chemical and photochemical stability [8,9]. The systematic control of nanostructure properties obtained by controlled variations in particle size and dimension is in direct contrast to molecular tags, whose properties vary nonsystematically between molecular species. This systematic variation of properties not only improves traditional applications, but also leads to new unique applications well beyond the scope of conventional molecular bioconjugates. The availability of these new nanostructures will greatly facilitate new *in situ* probes and sensor methods.

In the present work, the most significant applications of semiconductor nanostructures in the field of optical biosensing will be reviewed. In particular, the use of quantum dots as fluorescent bioprobes, which is the most widely used application, will be discussed. In addition, the use of some other nanometric structures in the field of biosensing, including nanoporous semiconductors and photonic crystals, will be discussed.

## 2. Semiconductor Quantum-Dot-Based Biosensors

The most common method of detecting and quantifying biomolecules still remains the use of fluorescence [5,7], which involves the use of fluorescent labels. The earlier classes of these labels included organic dyes, fluorescent proteins and lanthanide chelates, which are still commonly used mainly because of their small size, ease of usage and the existence of standard protocols for their bioconjugation. A vast library of fluorophores has been synthesized over time, many of which are designed for very specific applications. Accordingly, such fluorescent probes have found ample use in many different biosensing applications including immunoassays, nucleic acid detection, resonance energy transfer studies, clinical/diagnostic assays and cellular labeling [6,9].

However, many of the organic dye and protein-based fluorophores suffer from serious chemical and photophysical limitations caused by their intrinsic properties, which have limited their effectiveness in long-term stability and simultaneous detection of multiple fluorescent signals, i.e. multiplexing, without complex instrumentation and processing. Some drawbacks that can be highlighted include narrow absorption windows coupled to broad red-tailed emission spectra via small Stokes shifts, short excited state fluorescent lifetimes, pH dependence, self-quenching at high concentrations and susceptibility to photobleaching.

### 2.1. Properties of Semiconductor Quantum Dots Materials

**Figure 1.**
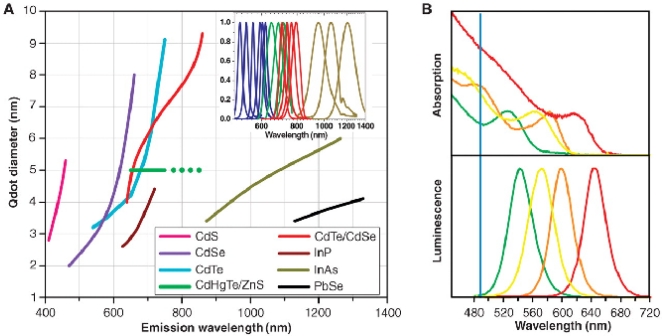
(A) Emission maxima and sizes of quantum dots of different composition. Quantum dots can be synthesized from various types of semiconductor materials (II–VI: CdS, CdSe, CdTe, etc.; III–V: InP, InAs, etc.; IV–VI: PbSe, etc.). The curves represent experimental data from the literature on the dependence of peak emission wavelength on quantum diameter. The range of emission wavelength is 400 to 1350 nm, with size varying from 2 to 9.5 nm. All spectra are typically around 30 to 50 nm (full width at half maximum). Inset: Representative emission spectra for some materials. (B) Absorption (upper curves) and emission (lower curves) spectra of four CdSe/ZnS quantum dot samples. The blue vertical line indicates the 488-nm line of an argon-ion laser, which can be used to efficiently excite all four types of quantum dots simultaneously. Reproduced from [10].

The unique fluorescent and overall optical properties of semiconductor quantum dots (QDs or semiconductor nanocrystals), make them very interesting fluorophores for both *in vivo* and *in vitro* biological investigations [10, 11]. Semiconductor nanocrystals are highly light absorbing and luminescent nanoparticles whose absorbance onset and emission maximum shift to higher energy with decreasing particle size (Figure 1) due to quantum size effects. Thus, the wavelength of emission can be tuned by altering their size (and chemical composition), giving rise to a wide spectrum of emission colors. Compared with molecular dyes, two properties in particular stand out: the ability to size-tune fluorescent emission as a function of nanocrystal size and the broad excitation spectra. The systematic control of the properties of QDs is in direct contrast to molecular tags, whose properties vary nonsystematically between molecular species. The systematic variation of the physical properties of QDs via structure variation not only improves traditional applications, but also leads to novel and unique applications well beyond the scope of conventional molecular bioconjugates.

Quantum dots usually show symmetric and narrow (bandwidth of around 30 to 50 nm full width at half maximum) photoluminescence spectra spamming the ultraviolet to near-infrared, thus enabling emission of pure color (Figure 1). By contrast, the bandwidths of organic dyes (fluorescein for instance) typically vary between 50 and 100 nm. Unlike molecular fluorophores, which posses narrow excitation spectra, semiconductor quantum dots show broad absorption spectra, generally starting to the blue of the emission peak of the QD and increasing steadily towards the ultraviolet regardless of their size. QDs also have relatively high quantum yields (resulting in high brightness) and high resistance to photobleaching and chemical degradation. Also, the molar extinction coefficients of QDs are much larger than those of conventional organic dyes. This leads to large effective Stokes shifts, thus allowing to efficiently excite a mixed population of QDs at a single wavelength far removed (> 100 nm) from their cumulative emissions. This enables the use of QDs for multiplexing by probing several markers at a time with a single excitation source, thus preventing overheating of cells or tissue during multi-color imaging, leading to great promise for both *in vitro* and *in vivo* applications and to simplification in instrumental design. This feature can be hard to achieve with conventional fluorophores due to their overlapping absorption and emission spectra. Photoluminescence lifetimes of QDs are usually long, which allows imaging of living cells without interference from background autofluorescence. All these issues, together with stability (much less photodestruction) and large surface-to-volume ratios, make QDs superior to organic fluorophores in detection sensitivity as well as in long-term tracking of biological processes. Cumulatively, these fluorescent properties will lead to the creation of a new generation of robust biosensors.

Furthermore, the possibility of tuning the emission from the QDs as to improve spectral overlap with a particular acceptor dye, make QDs suitable for their use as efficient fluorescence resonance energy transfer (FRET) donors. Moreover, QDs also emit light at a rate slow enough to eliminate most of the autofluorescence in the background but fast enough to maintain a high photon turnover rate. Therefore, they are ideal probes for timegated detection with enhanced selectivity and sensitivity. Additionally, it is possible to obtain polarized fluorescence by using shape-controlled QDs.

However, although possessing superb optical characteristics, QDs undergo an intermittent on–off emission under continuous excitation. This property, called blinking, is only partially understood and has been attributed to Auger ionization, caused by fluctuations in net charge inside or around the nanocrystals [12]. The effect of chemical environment has been demonstrated to dramatically influence the fluorescence blinking dynamics. Blinking might be a concern when a signal from individual QDs is required during the analysis. However, in general, for example in cell-based assays, there are more than one quantum dot involved and, while some are *blinking*, others can be *on* for the final detection and thus no signal will be missed by the detector. For single particle tracking, the irregular blinking of quantum dots is a minor drawback. Table 1 presents an overview of several properties of QDs compared to those of traditional fluorophores.

The fabrication techniques for the growth of semiconductor nanocrystals have been extensively developed in the past few decades, mainly focused on traditional applications in optoelectronic devices. These techniques allow the size, shape and composition of the nanocrystals to be varied over a remarkably wide range. Generally speaking, semiconductor QDs for applications in the field of optical biosensing are synthesized with mostly direct-band-gap materials (II–VI or III–V column elements of the periodic table). Accordingly, quantum dots made of ZnS, CdS, ZnSe, CdTe and PbSe, emitting from the UV to the infrared have been prepared for bioapplications, although these may need refinement as issues of reproducible synthesis and inorganic passivation remain to be optimized.

### 2.2. Core-Shell Quantum Dots

There are two major problems associated with the stability of luminescence from semiconductor nanocrystals. The first one is related to the presence of surface states arising from surface non-stoichiometry and unsaturated bonds. Crystalline imperfections and defects found on the surface of QDs will capture excited state energy and provide nonproductive and non-emissive pathways for deactivating the QD after they have been excited with light. Secondly, the surfaces of uncapped QDs tend to be very reactive due to their high surface-area-to-volume ratio, and consequently they become easily polluted by a variety of agents, which in turn provide additional trapping pathways that also result in quenched emissions. At the same time, uncapped QDs are so reactive that they are prone to spontaneous dissolution or photochemical degradation. Hence, even the simple act of diluting QDs often leads to irreversible decomposition of the nanocrystals. Therefore, control of the surface properties is mandatory for the formation of highly luminescent and chemically stable QDs. This issue has led to the development of core-shell quantum dot structures with improved properties [13].

Core-shell geometries where the nanocrystal is encased in a shell of a wider band gap semiconductor have resulted in increased fluorescence quantum efficiencies (over 50%) and greatly improved photochemical stability [11]. As an example, in the visible region, CdSe-CdS core-shell nanocrystals have been shown to span the visible region from 550 nm (green) to 630 nm (red). CdTe QDs, with typical emissions ranging from 650–850 nm, have been less common in biosensing applications mainly due to relatively broad emission spectra. Other materials, such as InP and InAs, provide QD fluorophores in the near infrared region of the optical spectrum, a region of high physiological transmissivity. However, the best available QD fluorophores for biological applications are made of CdSe cores overcoated with a layer of ZnS. The ZnS layer passivates the core surface, protects it from oxidation, prevents leeching of the Cd/Se into the surrounding solution and also produces a substantial improvement in the photoluminescence yield. Even though thin ZnS (1–2 monolayers) shells often produce the highest photoluminescence yields, thicker ZnS shells (4–6 monolayers) provide more core protection against oxidation and the harsher conditions presented by biological media.

As a result of their superior optical properties QDs are being increasingly used for *in vivo* applications. The recent upsurge of *in vivo* studies has proved that QDs are just as effective here as in the test tube. Successful imaging of live cells with semiconductor QDs as labels has further promoted the popularity of using QDs in biological systems. However, biotoxiticy of such elements as Cd could be a major concern for *in vivo* applications. This question has been investigated by a number of groups [10,11]. In these studies it has been reported that surface oxidation can occur under combined exposure to the aqueous/ultraviolet-light excitation, which can lead to the release of cadmium ions in the case of CdSe-based QDs. The surface oxidation of the core QDs can be reduced by the use of core-shell structures, which could create a barrier for oxygen diffusion. However, a combined aqueous/ultraviolet-light excitation environment can still act as a catalyst and enhance the diffusion process. Before QDs are adopted for *in vivo* applications, a comprehensive study of shell type and thickness, as well as the relative diffusion rate of oxygen need to be well understood.

**Figure 2.**
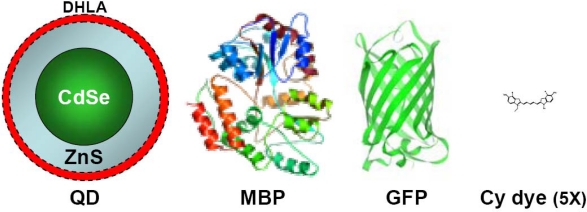
Comparison of the size of a representative dihydrolipoic acid (DHLA) capped CdSe/ZnS QD (550 nm emission and diameter ∼ 6 nm), to a maltose binding protein (MBP) molecule (mw∼ 44,000), green fluorescent protein (GFP, mw∼30,000) and a cyanine dye (Cy, mw∼700). Reproduced from [11].

The typical physical size of core-shell QDs is almost an order of magnitude larger than many of the conventional organic dyes in use (Figure 2). For instance, CdSe-ZnS core-shell materials can range in size from 2 nm diameter (emission at 480 nm) to 8 nm (emission at 660 nm) while the redder CdTe-CdSe nanocrystals can range from 4 nm diameter (emission at 650 nm) to over 9 nm (emission at 850 nm) [11]. Redder emitting QDs tend to be anisotropic and can have large aspect ratios. Anyhow, the typical size of QDs makes it possible to introduce colloidal quantum dots into cells. With increasing demand for imaging structures deep inside the body, there is an increased interest in QDs emitting in the near-infrared region (approximately from 650 to 1,000 nm), a region where transmission of light through tissues and blood is maximal. Theoretical studies show that long-wavelength adsorption by biological tissue minimizes the background noise, since cellular autofluorescence is greatly reduced. QDs with tunable photoemission in the near infrared range, such as HgTe, CdHgTe, PbSe, InP, and InAs have been successfully prepared for biological applications. Thus, semiconductor QDs can be considered new and robust fluorophores, absorbers and scatterers in the near infrared, a region of the electromagnetic spectrum where tissue is essentially transparent.

### 2.3. Bioconjugation of Quantum Dots: Inorganic–Biological Hybrids or Hybrid Bioconjugates

Since highly luminescent QDs are usually fabricated from organometallic precursors and salts they have no intrinsic aqueous solubility, are often non-biocompatible, and do not have any reactive functional groups for conjugation with biomolecules [6,7]. The surface of the QDs must be functionalized with a ligand that can impart both solubility and potential bioconjugation sites. Inorganic-biological hybrids are made by conjugating inorganic nanostructures with biomolecules (proteins, DNAs). Thus, the resulting hybrids combine the properties of both materials, i.e., the spectroscopic characteristics of the nanocrystals and the biomolecular function of the surface-attached entities.

In this regard, the typical size of the QDs, comparable to or slightly larger than that of many proteins (Figure 2), needs not be considered a restriction for many applications as can provide several inherent benefits. Multiple proteins, peptides or other chemical moieties can be attached to a single QD. The QD thus acts as a nanoscaffold for the attachment of several biomolecules, creating a multifunctional nanoparticle-biological hybrid. Each biomolecule can impart some unique property to the resultant QD-conjugate, thus engendering multi-functionality. Alternatively, attaching multiple biomolecules to a QD can increase the avidity and help lower the limit of detection.

Colloidal quantum dots with a wide range of bio-conjugation and with high quantum yields are now available commercially. The range of biological experiments that these materials are employed in is rapidly growing, being this one of the first commercial applications of modern nanotechnology. Specific binding to cell surfaces, insertion into cells, and binding to cell nuclei or other organelles have all been demonstrated following conjugation of the nanoparticle with the appropriate targeting protein. A range of biomolecules, including deoxyribonucleic acid (DNA) and proteins have been conjugated to QDs and used in diverse biomedical studies such as *in vitro* detection assays, deep tissue imaging, and most recently in the selective and generalized imaging of living cells and organisms.

### 2.4. Cell Internalization

Several aspects of biological relevance regarding the use of QDs emerge when a novel biomedical application is envisaged. These aspects include stability, toxicity and internalization mechanism, which are in many cases interrelated. All these potential sources of conflict can be well focused by appropriate surface biofunctionalization to obtain bioconjugates composed of QD nuclei with surrounding biomolecules based on amino-acid or nucleic-acid sequences. Furthermore, with an efficient surface binding of such molecules one can exploit the specific recognition properties of proteins and oligonucleotides opening the gate to preferential site organization of the QDs in the cell.

Cell viability studies are mandatory steps before application of any QD-complex [14]. Results should provide a certain range of cell resistance to the potential damaging effects of QDs. These ranges vary in the literature from minimal damage within the time of cellular uptake [15], to viability after multiple cell divisions providing long term fluorescence [16]. There is a general agreement in that, given a biofunctionalization process, cellular fate depends on physical properties of the QD based complexes [17]. Cytotoxicity can be detected by cell expression. For instance, cytotoxicity in neuroblastoma cells occurs through the increased regulation of Fas, a receptor inducing apoptosis [18]. Previous steps before cell death may be indicated by accumulation of QDs in endosomes [14,15] or smaller vesicles [19].

Internalization of QDs in the cells can take place in diffusive and active forms [14,20], which depend dramatically on the type of cell and the biofunctionalization scheme of the QDs. Among the most referenced methods for a detailed study of this internalization are confocal microscopy [21] and flow cytometry [22]. Related studies show that diffusive processes taking place to reach osmotic equilibrium are not efficient enough and should be enhanced by active receptor mediated endocytotic pathways [23,24]. For this latter point, biofunctionalization with carrier molecules is a key issue. These membrane access molecules can be in the form of a branched peptide [14], an oligoarginine sequence [15], or a vasoactive intestinal peptide [25]. The mechanism to fix such peptides is very frequently mediated by a biotin-streptavidin linkage involving association of the peptide to biotin and the QDs to streptavidin [26,27].

Alternative methods for biofunctionalization exist based mainly in the interaction of the QDs with lipid layers, bilayers and nanomicelles conveniently linked to antibodies or other kind of proteins [23]. Lipids can be combined or substituted with poly(ethylene-glycol) (PEG) [28] specially in *in vivo* assays where capture in liver is to be avoided (increased plasma terminal half life). PEG chains allow also subsequent binding to a protein through amino terminal groups in the chain [29]. Among interesting proteins linked to QDs we can mention some with membrane access role such as transferring [24] though conjugation at the aminoacid scale has been also reported to be effective with histidine [15] and cysteine [18], both showing metal affinity interactions and thus prone also to direct bonding to QDs. Relevantly, gastric administration of gluthathione-QD conjugates has been proved to be effective to induce QD fluorescence in the endoderm of invertebrates [30].

With such a large choice of biofunctionalization possibilities, QD cell internalization studies are currently merged in an intensive development. Studies include QD evaluation in both eukaryotic and prokaryotic [26] species (in the virus case labeling with QDs does not imply internalization but tagging). In the former case, examples can be found for healthy living cells [14] including neurons [19,31] and epidermal cells [21,27]. Combinations for labeling have developed to the point of creating more than 100 codes for mammalian cells allowing the identification of complex phenotypes in mixed cellular populations [22]. In several cases conjugated QDs have been also the subject of studies with tumor cells [18,23,28]. These studies are becoming increasingly relevant since QDs are becoming not only an imaging tool, but also an active cancer therapeutic vehicle by allowing controlled drug delivery [14,23,25].

Examples of the degree of development of applications by using internalized QD conjugates include *in vivo* imaging capability [20,23] even in the infrared wavelength [29] used as a diagnostic or fundamental research tool. In fact QDs have become a useful element to reveal neuronal enclocytic events by conjugation with nerve growth factor [20]. Other fundamental aspects related to retrograde axonal transport have been studied by using the same nerve growth factor based complexes [19,31]. A final example of application of conjugated QDs consists in the possibility to deactivate QDs by including a fluorescence inhibitor (for instance a quenching molecule interfering electron excitation). This inhibitor is cleavable due to disulfide bond liberation upon cell internalization [29] in such a way that reduced background signal is obtained from non internalized QDs (more than 85%) fluorescence inhubition.

### 2.5. Applications

QDs are not meant to replace fluorescent dyes and proteins, but rather to be a specialized tool that can augment and complement them. However, several associated areas still need to be developed, principally the surface functionalization ligands and the related methods for conjugating QDs to biomolecules (especially orthogonal conjugation). As these mature, QDs are expected to become a more versatile tool for all aspects of fluorescent biosensing. In the following sections some particular examples of the use of quantum dots in the field of biosensing will be shown.

#### 2.5.1. Immunoassays/Multiplexing

**Figure 3.**
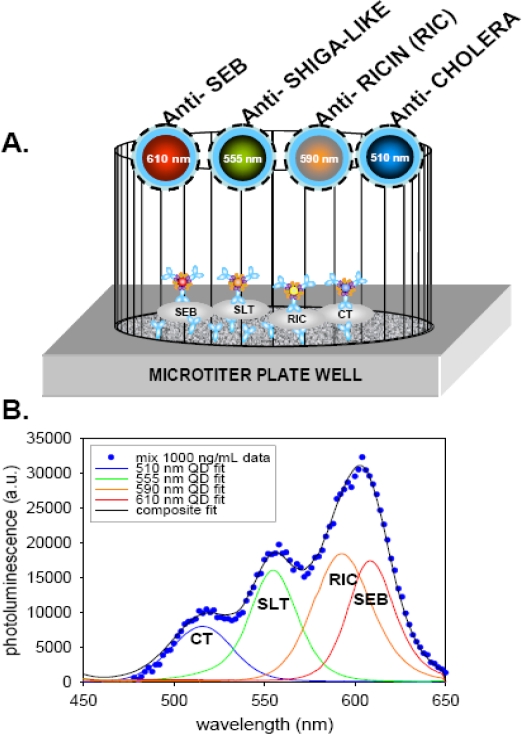
(A) Schematic of a 4-color multiplex assay. The indicated colors of QDs were prepared with antibodies against the 4-indicated toxins and simultaneously incubated in microtiter-well plates containing the 4-toxins immobilized by capture antibodies on the surface. (B) Multi-toxin assay examining mixes of all four indicated toxins at 1000 ng/mL each probed with a mix of QD-detection antibody conjugates. Measured values are shown as circles. Both the composite fit and the fit from each of the four individual QD components are displayed. Reproduced from [11].

The unique advantages that QDs offer over conventional dyes has increasingly led to their use in immunoassay detection. For biosensing, the greatest potential of QD-antibody bioconjugates is in multiplexing [32]. In a demonstration of this potential, immunoassays based on QDs were used for the simultaneous detection of four toxins: cholera toxin, ricin, shiga-like toxin 1 and staphylococcal enterotoxin B (SEB), in a single microtiter well (Figure 3). In this assay, capture antibodies immobilized in a microtiter well plate were first exposed to the mixed toxin sample. Antibodies specific for each of the toxins coupled to a different color QD were then added to the microtiter well plate. The resulting signal from the mixed toxin samples was then deconvoluted using a simple algorithm. In another example, QD-antibody bioconjugates were used to identify and differentiate between diphtheria toxin and tetanus toxin proteins which were non-specifically immobilized onto poly-L-lysine coated cover slips. Additionally, the simultaneous detection of Escherichia coli O157:H7 and Salmonella Typhimurium bacteria using different colored QDs as immunoassay labels has been successfully demonstrated. While these studies represent only initial proof-of-principle, and further optimization and refinement will be required to improve limits-of-detection, they clearly demonstrate the potential of QDs in multiplexed immunoassay formats. The only major obstacle to future 6 to 10 color QD multiplex immunoassays still remains the inherent cross-reactivity of antibodies.

#### 2.5.2. Electrochemical Detection

**Figure 4.**
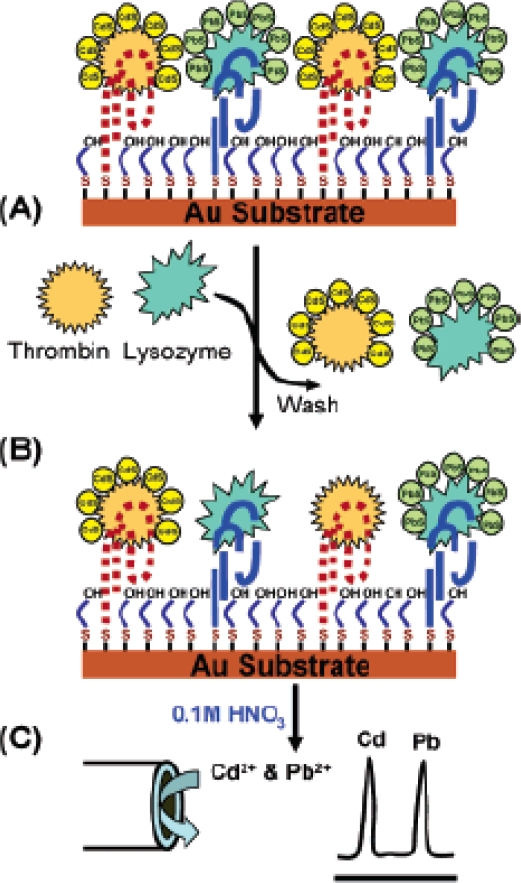
Operation of the aptamer/QD-based dual-analyte biosensor, involving displacement of the tagged proteins by the target analytes. (A) Mixed monolayer of thiolated aptamers on the gold substrate with the bound protein-QD conjugates; (B) sample addition and displacement of the tagged proteins; (C) dissolution of the remaining captured nanocrystals followed by their electrochemical-stripping detection at a coated glassy carbon electrode. Reproduced from [33].

Semiconductor nanocrystals can be used as to increase efficiency of photochemical reactions and can be effectively coupled to biomolecular units such as enzymes, to generate novel photoelectrochemical systems. Within this approach two broad strategies have been followed involving the use of the electrochemical properties of QDs for biosensing: (i) the monitoring of QD electrochemistry directly; or (ii) monitoring of QD photoelectrochemistry. As an example of the first approach, a multiple protein aptamer-based biosensing capability is coupled to the enormous amplification feature of nanoparticle based electrochemical stripping measurements to yield remarkably low detection limits [33]. This is accomplished using a simple single-step displacement assay (Figure 4), involving the co-immobilization of several thiolated aptamers, along with binding of the corresponding QD-tagged proteins on a gold surface (Figure 4A), addition of the protein sample (Figure 4B) and monitoring the displacement through electrochemical detection of the remaining nanocrystals (Figure 4C). Such electronic transduction of aptamer-protein interactions is extremely attractive for meeting the low power, size and cost requirements of decentralized diagnostic systems. Unlike two-step sandwich assays used in early QD-based electronic hybridization or immunoassays, this aptamer biosensor protocol relies on a single-step displacement protocol.

#### 2.5.3. Sensing Based on Fluorescence Resonance Energy Transfer (FRET)

One method for using QDs in biosensing is to create a donor/acceptor complex, which exhibits switching capability via fluorescence resonance energy transfer (FRET). FRET between donor and acceptor molecules has been extensively used in biophysical and biochemical studies to probe ligand-receptor binding and molecular structural changes. This is directly attributable to the sharp efficiency dependence of the process on the donor-acceptor separation distance at the 1–10 nanometer scale. QDs offer several advantages when used as FRET donors in place of organic dyes [34,35]. The possibility of tuning the emission spectra together with its typical narrowness can considerably reduce donor spectral leakage into the acceptor channel. At the same time, their broad absorption spectrum at wavelengths to the blue of their emission allows choice of excitation that corresponds to the acceptor absorption minimum, substantially reducing direct excitation. While QDs are not expected to replace organic dyes in all FRET applications, recent studies suggest that they might significantly improve assay performances in a wide variety of sensing schemes [34]. However, the main limiting factor in the performance of QDs as FRET donors lies with their size because the FRET efficiency depends on the center-to-center separation between donor and acceptor. Generally speaking, three variables contribute to overall QD donor size, namely the core shell radius, the particular coating and the bioconjugation strategy.

In this regard, several studies have demonstrated the effective use of QD FRET donors to detect small analytes by utilizing a common strategy that relies on conjugating QDs to target binding receptors which can be either proteins, antibody fragments or DNA aptamers. The QDs conjugates are then exposed to appropriate acceptor-labeled target analogs which are brought in close proximity to the QDs by binding to the receptors. In this initial state, the QD-donor photoluminiscence is quenched by efficient FRET to the proximal acceptor-labeled analogs. The presence of the target then displaces bound analogs from the surrounding conjugated receptors and this is detected through a reduction in FRET efficiency and the concomitant QD photoluminescence increase. This strategy has been utilized for detecting diverse analytes. Overall, this type of detection method benefits from a wide library of receptor proteins, antibodies and aptamers which provide both flexibility and specificity. However, it requires the presence of labeled analogs in solution, and is therefore not suitable for continuous monitoring. However, it is worth noting that some of the same optical properties that make QDs excellent FRET donors may also hinder their use as FRET acceptors. While their broad absorption spectrum and high excitation cross sections result in large spectral overlaps and high FRET efficiencies, this can also result in the unavoidable direct excitation of the QD acceptor at a rate that is often greater than the FRET induced excitation. In addition, the QDs longer exciton lifetime compared to that of many organic dyes may also hinder efficient FRET from dyes to QDs.

**Figure 5.**
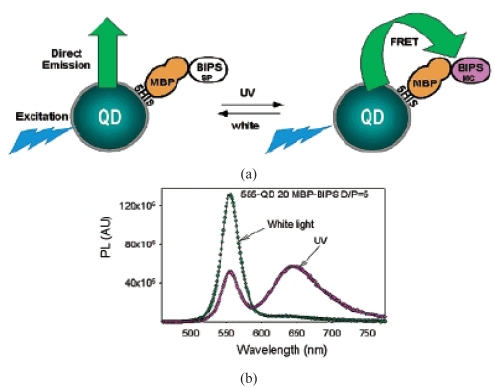
(a) Schematic of QD modulation by MBP-BIPS. (b) Spectral properties and modulation function of MBP-BIPS and the 555 nm emitting QDs. Reproduced from [36].

An interesting biosensor showing reversible FRET has been fabricated by connecting CdSe/ZnS core/shell QDs with a photoactivatable species that functions as the reversible FRET acceptor [36]. QDs are connected to photochromic 1′,3-dihydro-1′-(2-carboxyethyl)-3,3-dimethyl-6-nitrospiro-[2*H*-1-benzopyran-2,2′-(2*H*)-indoline] (BIPS) via a bridge of maltose binding protein (MBP) (Figure 5a). Exposure to ultraviolet light catalyzes the photoconversion of BIPS from the colorless spiropyran to the colored merocyanine form, which functions as the FRET acceptor and therefore modulates QDs emission. The photoconversion is reversible, with white light converting merocyanine back to the spiropyran form. Quenching of QD emission at 555 nm and enhancement of BIPS emission at 650 nm via FRET appears upon exposure of the complex to ultraviolet light (Figure 5b). Well-controlled, reversible switching events were demonstrated by alternating the illumination light source between white and ultraviolet light. Incorporation of an emission unit that can be modulated via a biological stimulus enables the creation of photochromically switched devices or sensors, where QD emission modulation presets the device below some predetermined critical threshold. The detection limits of analytical processes based on FRET can be as low as 10 ppt with a linear dynamic range from 0.1 ppt to 1,000 ppt. For the purpose of sensors, FRET efficiency could be enhanced further by using luminescent nanostructures with high surface-to-volume ratios. These advances could lead to powerful, compact sensors.

Additionally, the optical properties of QDs can be exploited as to improve the assay sensitivity of single particle DNA sensing. Incubation of dye-labeled DNA targets with biotinylated capture DNA probes allows their conjugation to streptavidin QDs only when the two DNA sequences hybridize. This hybridization is then detected via FRET between the QD and the dye acceptor. This approach utilizes commercial QD materials and their large size (∼30 nm diameter) initially suggested that relatively poor FRET efficiency would result. However, in this particular case, the high QD quantum yield (≥ 50%), the high Cy5 acceptor molecular extinction coefficient (∼250,000 M^−1^ cm^−1^) and a large number of acceptors (typically 12 to 54) around each donor can combine to overcome distance constraints arising from the QD size. Additionally, the background due to acceptor direct excitation is virtually eliminated through the choice of an appropriate excitation wavelength. This led to a 100-fold improvement in sensitivity compared to single organic dye molecular beacon-based detection. These type of sensing schemes can also be adapted for their use in a multiplex format. The narrow and symmetric QD emissions allowing easy spectral deconvolution and the most straightforward configuration relies on several QD populations interacting with the same dye acceptor, rather than the opposite.

**Figure 6.**
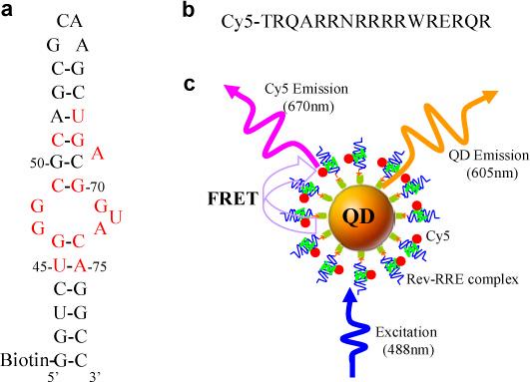
The principles of a QD-based nanosensor for Rev-RRE interaction assay. (a) Secondary structure of biotinylated RRE IIB RNA. Nucleotides identified as important for Rev binding are shown in red. (b) Sequence of Cy5-labeled Rev peptide. (c) Conceptual scheme of the QD-based nanosensor for Rev-RRE interaction assay based on FRET between 605QD and Cy5. The binding of a Cy5-labeled Rev to a biotinylated RRE IIB RNA formed a Rev-RRE complex, which was caught on the surface of a 605QD to form a 605QD/Rev-RRE/Cy5 assembly through specific streptavidin-biotin binding. FRET occurred between the 605QD and Cy5 upon illumination of the 605QD/Rev-RRE/Cy5 assemblies with an excitation wavelength of 488 nm. Reproduced from [37].

Figure 6 shows the principles of a QD-based nanosensor for Rev-RRE interaction assay [37]. Rev is an important HIV-1 regulatory protein that binds the Rev responsive element (RRE) within the env gene of HIV-1 RNA genome; the binding of Rev to RRE is essential for the expression of the structural genes gag-pol and env, and for HIV replication.

#### 2.5.4. Nanobarcodes

Multicolor optical coding for biological assays has been achieved by embedding different-sized quantum dots, such as zinc sulfide–capped cadmium selenide nanocrystals, into polymeric microbeads at precisely controlled ratios [38]. This approach can be used to create a large spectrum of beads with different colors and intensities for multiplexed, high throughput screening of DNA or proteins, thus using the advantages of QDs over organic dyes. The spectrum of QD-embedded microbeads has been reported to be 10% narrower than the QDs alone, which further benefits the multiplexed imaging. These promising microbeads were applied for DNA detection and hybridization of target sequences. They can withstand higher temperatures during the hybridization process than QDs. The sensitivity to the low amount of target sequences has not yet been determined. DNA hybridization studies demonstrate that the coding and target signals can be simultaneously read at the single-bead level, implying the potential of this coding technology in gene expression studies, high throughput screening, and medical diagnostics [39].

**Figure 7.**
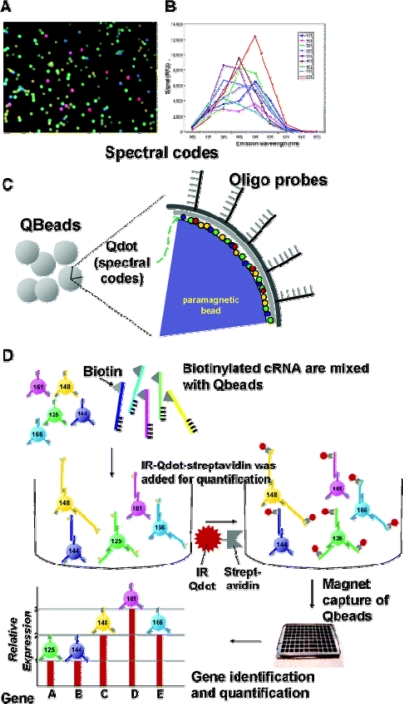
Schematics of QD nanobarcoded microbead system for high-throughput gene expression analysis. (A) Pseudocolor picture of the microbeads embedded with quantum dots. (B) Example spectra of the beads coded with different mixture of QDs. (C) Construction of the nanobarcoded microbeads. Each bead has a distinctive ratio of four different QDs, allowing identification by a characteristic spectral nanobarcode. The transcript-specific oligonucleotide probes are conjugated to the bead surface. Therefore, each spectral-barcoded bead detects a specific oligomucleotide determined by the probe. (D) Gene expression monitoring and quantification sandwich assay. The nanobarcoded microbead-attached oligo probes capture biotinylated cRNA sample through hybridization, the cRNA is further sandwiched by the 655 nm streptavidin QDs (or 705 nm, 800 nm) to be quantified. Reproduced from [39].

Figure 7 shows a nanobarcoded bead platform that can not only identify but also accurately quantify the gene expression variations in a high- throughput and multiplexed format, using 8-μm-diameter magnetic beads. Using four colors, 455 genes can be theoretically monitored at the same time.

As such, these recent investigations certainly open up vast opportunities for creating a new generation of fluorescent markers with immense promise and potential in biological assay and detection. However, despite some breakthrough advances in the synthesis of QD-tagged latex beads and their applications to multiplexed optical encoding, there remain many problems yet unresolved. The main challenges that must be addressed in the use of these nanobeads in biological applications are how to better control the incorporation of the QDs into the polymer particles, as well as the control of colloid stability and monodispersity. More recently, work has also been done in the area of incorporating QDs into biocompatible polymer beads.

## 3. Porous Nanostructures for Biosensing

In addition to nanoparticles, novel materials such as porous silicon (PS) and porous carbon with nanometric pore size, compatible with the dimension of biomolecules have been used for biosensor applications. Other nanoporous thin films such as zinc oxide have also been used.

Since the discovery of its strong visible luminescence at room temperature, nanostructured porous silicon has attracted considerable interest aiming at the fabrication of optoelectronic devices, including biosensors. Additional interest is given to PS since this material can be fabricated easily using some of the established processes of the usual silicon technology, by partial electrochemical dissolution in hydrofluoric acid-based solutions, thus leading to controlled morphology at a nanometer scale in three dimensions. Furthermore, its large surface area allows quite easy chemical surface modification thus allowing the development of sensitive biosensors [40,41]. The biocompatibility of PS can be improved by a suitable change of the fabrication parameters.

**Figure 8.**
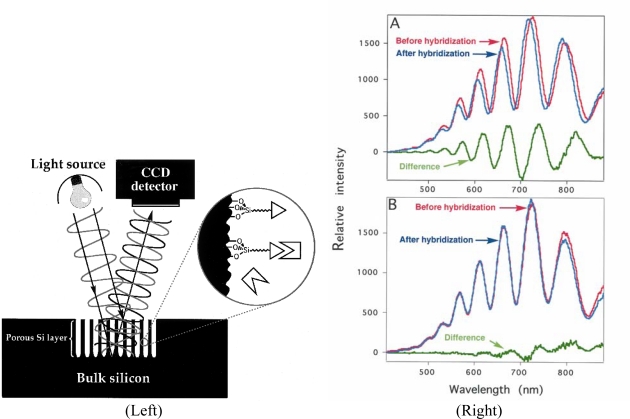
(Left) Schematic of a PS-based optical interferometric biosensor. The silicon oxide surface of the porous layer can be modified to express various molecular recognition elements (such as oligonucleotides, biotin or antibodies). Reflection of white light at the top and bottom of the PS layer results in an interference pattern (Fabry-Perot fringes). Interactions of the molecular species with their recognition partners immobilized on the surface induce a change in the refractive index of the nanocrystalline semiconductor, giving rise to wavelength shifts in the fringe pattern that can be easily detected (charge-coupled device (CCD) camera) and quantified. (Right) Interferometric reflectance spectra of DNA-modified PS layers. Experiments were performed for two DNA sequences and the corresponding complementary strands. For clarity, only one set of data is shown in each case. (A) The Fabry-Perot fringes from a PS surface derivatized with DNA-A (“before hybridization,” red trace) shift to shorter wavelength upon exposure to a 2 × 10^−12^ M solution of DNA-A’ (the cDNA sequence to DNA-A) in 1 M NaCl(aq) (“after hybridization,” blue trace). The net change in effective optical thickness (from 7,986 to 7,925 nm) upon DNA-A’ recognition is represented by the difference between the two interference spectra (“difference,” green trace). (B) The control experiment, showing the Fabry-Perot fringes of a DNA-A–derivatized PSi surface before and after exposure to a 2 × 10^−12^ M solution of DNA-B (non-cDNA sequence) in 1 M NaCl(aq). No wavelength shift was observed up to the measured concentration of 10^−9^ M DNA-B. Reproduced from [42].

PS-based biosensors based on optical interferometry have been developed, allowing the detection of small organic molecules (biotin and digoxigenin), 16-nucleotide DNA oligomers and proteins (streptavidin and antibodies) at pico- and femtomolar analyte concentrations [42]. The operational principle is based on induced wavelength shifts in the Fabry-Perot fringes in the visible-light reflection spectrum of appropriately derivatized thin films of porous silicon semiconductors. Binding of molecules induced changes in the refractive index of the porous silicon. Accordingly, in the presence of complementary DNA (cDNA) sequences, pronounced wavelength shifts in the interference pattern of the PS thin films are induced (Figure 8). Under similar conditions but in the presence of non-cDNA sequences, no significant shift in the wavelength of the interference fringe pattern is detected (only minor amplitude fluctuations are observed). This type of optical biosensor has also been demonstrated to be highly effective for detecting single and multilayered molecular assemblies. In addition to these estructures, microcavity resonators made of PS have been used as biosensors and demonstrated in DNA detection. Such microcavity structures are highly sensitive and any slight change in the effective optical thickness modified its reflectivity spectrum, causing a spectral shift in the interference peaks. This makes porous silicon microcavities an ideal host for biosensor applications.

**Figure 9.**
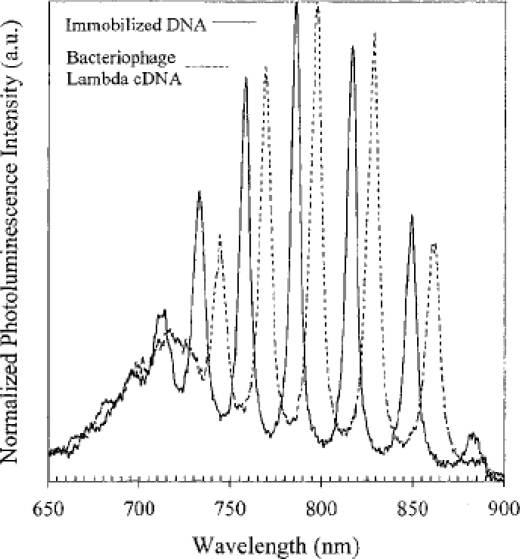
Recognition and binding of bacteriophage lambda DNA to a 30-nucleotide complementary DNA sequence immobilized on a silicon chip is detected by a 12 nm red-shift in photoluminescence. The DNA concentration of the sensing bacteriophage lambda is 194.2 fM. Reproduced from [43]. Copyright Wiley-VCH Verlag GmbH & Co. KGaA. Reproduced with permission.

Furthermore, PS-based microcavities have been developed for their use as biosensors, by exploiting the luminescent properties of PS [43]. Porous silicon microcavity resonators possess the unique characteristics of line narrowing and luminescence enhancement. The emission peak position can be tuned by changing the properties of the central layer. Increasing the thickness of the central active layer introduces multiple narrow peaks in the luminescence spectrum. Narrow and visible luminescence peaks are observed with a full width at half the maximum (FWHM) value of 3 nm. The usefulness of microcavity resonator structures as biosensors was demonstrated by fabricating a DNA biosensor based on a PS multiple peak microcavity structure. Shifts in the luminescence spectra are observed and detected for various DNA concentrations. When exposed to a non-complementary DNA strand, no shifts are observed. An extension of the DNA biosensor can be made to include the detection of viral DNA. Thus, the recognition and binding of bacteriophage lambda to a partial c-DNA sequence immobilized in the porous matrix can be confirmed through photoluminescence spectral shifts. Figure 9 shows a 12 nm red-shift in the photoluminescence peaks, induced by a change in the effective refractive index of the material upon coupling of the nucleotides. A relatively large red-shift is observed.

Finally, potentiometric biosensors based on porous silicon have been described [44]. The enzymes penicillinase and lipase were separately immobilized on the surface of porous silicon to detect penicillin and triglycerides. The hydrolysis reactions caused a change in the pH of the solution. The enzyme solution-oxidized porous silicon-crystalline silicon structure was used to detect the changes in pH during hydrolysis as a shift in the capacitance–voltage (C–V) characteristics.

One of the most common materials used as matrices for the design of biosensor systems is carbon. Graphite, glassy carbon and carbon paste are some of the carbon forms that have been used so far for biosensor development [ 45 ]. Porous conductive carbon has been utilized for the successful development of biosensors, since this material has shown to be a good matrix for the reproducible construction of these devices. The high conductivity of this carbon material is ideal for the electrochemical signal transduction, while at the same time its high porosity allows the adsorption of large molecules. Porous conductive carbon has been used with great success for the construction of highly stable and reproducible glucose and lactate biosensors. In particular, the activated carbon matrix has been used for the immobilization and stabilization of the enzyme acetylcholinesterase. The enzyme is immobilized by adsorption into the nanostructured conductive carbon, which also acts as the working electrode of the biosensor. Using this biosensor, the monitoring of the organophosphorus pesticide dichlorvos at picomolar levels has been achieved in the inhibition mode.

Glassy carbon is another material that has been well established for many years as an electrode material and more specifically as a biosensor immobilization matrix. Structurally, it is a compact solid, mechanically stable, impermeable to gases and fluids and chemically resistant over a broad range of situations. In addition, the high porosity, the low background current over a wide potential range and the great applicability of this material make it ideal for the adsorption of large molecules, while at the same time suggest its possible use as an electrochemical transducer. Recently, novel material processes allow for the formation of porous glassy carbon, leading to the formation of micro, meso or macro porous structures. One of the drawbacks of this material when used in biosensor design is its low sensitivity to peroxide, as well as to other mediators. In any case, porous glassy carbon can be considered an excellent transducer for amperometric measurements, as is the case of porous carbon, while providing cavities adequate for enzyme immobilization.

## 4. Photonic Nanostructures Based on Semiconductors

By analogy with the control of the density of electron states in semiconductor quantum wells, wires, and dots, it is possible to control the density of photon states by creating a medium with artificially designed regions of varying index of refraction, i.e., photonic crystals (PhCs). Thus, by controlling the patterns of materials on a length scale comparable with the wavelength of light, in one, two, and three dimensions, nanometric structures with designed optical characteristics can be artificially created [46,47]. Additionally, by embedding a biomolecule in a photonic crystal, it is possible to control the rates and directions in which molecules emit light. This is complementary to systems where electrons are confined, and in which the energy of the emission can be controlled. Many photonic band-gap structures can now be prepared by a variety of means, some of which are potentially compatible with the incorporation of biological molecules which show great promise in biological detection.

In general terms, photonic crystals are an attractive biosensing platform because they provide strong light confinement. Unlike many sensing platforms that utilize the interaction between the small evanescent tail of the electromagnetic field and the analyte, PhCs can be designed to localize the electric field in the low refractive index region (e.g., air pores), which makes the sensors extremely sensitive to a small refractive index change produced by bio-molecule immobilization on the pore walls. Moreover, by introducing a point defect into a PhC, defect states can be pulled down from the air band or up from the substrate band. The corresponding optical spectra show narrow transmission peaks inside the bandgap, whose precise position is determined by the refractive index of the pores. Thus, the presence of molecules inside the pores can be detected by monitoring a small spectral shift, especially if high-Q microcavities, which have been reported both theoretically and experimentally. However, protein recognition depends on the surface chemistry, thus, instead of filling up the pores and changing the ambient refractive index, the molecules coat the pore walls.

**Figure 10.**
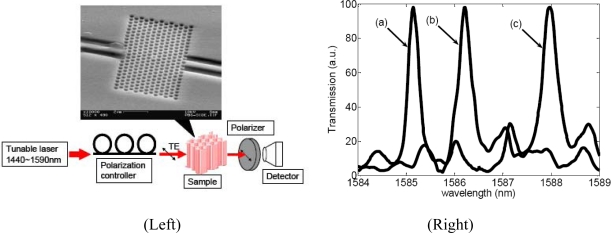
(Left) Scanning electron microscopy photograph of a typical photonic crystal and schematic of the experimental setup. A tunable laser (1,440 nm to 1,590 nm) is used as the source. Light is coupled in and out of the PhC using tapered ridge waveguides. A polarization controller is used to maximize the TE mode signal, and an InGaAs detector is used to measure the transmission signal. (Right) Normalized transmission spectra of the PhC microcavity. Curve (a) indicates the initial spectrum resonance after oxidation and silanization, curve (b) is measured after glutaraldehyde attaches to the pore walls, and curve (c) is obtained after infiltration of BSA molecules. Reproduced from [45].

A two-dimensional photonic crystal microcavity biosensor has been demonstrated [48] consisting of a hexagonal array of cylindrical air pores in a 400 nm-thick silicon slab separated from the silicon substrate by 1 μm of silicon dioxide to provide a good vertical confinement for the propagation modes. The PC has a lattice constant of 465 nm and a pore diameter of 270 nm. The defect is introduced by reducing the center pore diameter to 140 nm (Figure 10). Such a configuration gives rise to a resonance in the bandgap close to 1.58 μm for even (TE-like) modes. The device operates near its resonance at 1.58 μm. Coating the sensor internal surface with proteins of different sizes produces a different amount of resonance redshift. This device can detect a molecule monolayer with a total mass as small as 2.5 fg. The device performance has been verified by measuring the spectral resonance redshift associated with the binding of glutaraldehyde and bovine serum albumin. Its performance can be further improved by increasing the Q factor and positioning the biological substance in the defect region only. Experiments carried on specific biotin-streptavidin model indicate the selectivity of the device.

## 5. Conclusions and Future Perspectives

The use of quantum dots in the field of biosensing has been discussed in this review, including issues such as materials, bioconjugation of quantum dots and cell internalization. Additionally, the use of novel materials with nanometric pore size in the field of biosensing has been presented. Also, it was shown that photonic crystals are an attractive biosensing platform because they provide strong light confinement.

The current status of quantum dot technology is expected to evolve in a rapid fashion on many different aspects. The use of novel systems could lead to improved properties including narrower fluorescence emission and longer lifetimes, as well as suppression of blinking and quantum yield enhancement. Also, sensitivity to electric or magnetic fields may play an important role in future biosensor systems.

## Figures and Tables

**Table 1.** Comparison of organic/protein fluorophore and quantum dot properties. Adapted from [11].

**Property**	**Fluorophores**	**Quantum Dots**
**Photophysical**		
Absorption spectra	Variable/narrow generally a mirror of the emission spectra	Broad spectra, steadily increases towards the UV from the first absorption band edge
Molar extinction coefficients	Variable, generally < 200,000 M^−1^ cm^−1^	High, 10–100 times that of fluorophores
Emission spectra	Broad, asymmetric red-tailed emission	Narrow FWHM, typically 25 to 40 nm for CdSe core materials
Maturation time	Needed for fluorescent proteins	N/A
Effective Stokes shifts	Generally < 100 nm	> 200 nm possible
Tunable emission	N/A	Unique to QDs / can be size-tuned from the UV to IR
Quantum yield	Variable, low to high	Generally high, 0.2 to 0.7 in buffer depending upon surface coating
Fluorescent lifetime	Short < 5 ns	Long ∼ 10–20 ns or greater
Spectral range	Necessitates a different dye every 40–60 nm	UV-IR depending upon binary/ternary material Vis - CdSe
Photostability	Variable to poor	Excellent, strong resistance to photobleaching several orders of magnitude that of dyes
Multiphoton cross section	Variable to poor	Excellent > 2–3 orders of magnitude that of dyes
Single-molecule capabilities	Variable	Excellent
FRET capabilities	Variable, mostly single donor- single acceptor configurations	Excellent donors, size tune emission to improve the overlap with an acceptor dye, single donor-multiple acceptor configurations possible
Multiplexing capabilities	Rare	Excellent, largely unexplored
Intermittency (blinking)	Negligible	Maybe problematic in isolated circumstances (single molecule tracking)
**Chemical**		
Chemical resistance	Variable	Excellent
Reactivity	Multiple reactivities commercially available	Limited conjugation chemistries available
Mono-valent attachment	Easy	Difficult
Multi-valent attachment	Rare – mostly *bis*-functional	Good possibilities, can attach several molecules to QDs depending upon size
**Other**		
Physical size	< 0.5 nm	4 – 7 nm diameter for CdSe core material
Electrochromicity	Rare	Largely untapped
Cost effectiveness	Very good / multiple suppliers	Poor / few commercial suppliers
